# Microcystin-Detoxifying Recombinant *Saccharomyces cerevisiae* Expressing the *mlrA* Gene from *Sphingosinicella microcystinivorans* B9

**DOI:** 10.3390/microorganisms11030575

**Published:** 2023-02-24

**Authors:** Fernando de Godoi Silva, Daiane Dias Lopes, Ronald E. Hector, Maikon Thiago do Nascimento, Tatiana de Ávila Miguel, Emília Kiyomi Kuroda, Gisele Maria de Andrade de Nóbrega, Ken-Ichi Harada, Elisa Yoko Hirooka

**Affiliations:** 1 Department of Food Science and Technology, State University of Londrina, Londrina 86057-970, PR, Brazil; 2 Bioenergy Research Unit, National Center for Agricultural Utilization Research, USDA—Agricultural Research Service, Peoria, IL 61604, USA; 3 Department of Civil Engineering, State University of Londrina, Londrina 86057-970, PR, Brazil; 4 Department of General Biology, State University of Londrina, Londrina 86057-970, PR, Brazil; 5 Graduate School of Environmental and Human Science, Meijo University, Nagoya 468-0073, Japan; 6 Faculty of Pharmacy, Meijo University, Nagoya 468-0073, Japan

**Keywords:** microcystinase, heterologous expression, biodegradation, public health

## Abstract

Contamination of water by microcystins is a global problem. These potent hepatotoxins demand constant monitoring and control methods in potable water. Promising approaches to reduce contamination risks have focused on natural microcystin biodegradation led by enzymes encoded by the *mlrABCD* genes. The first enzyme of this system (*mlrA*) linearizes microcystin structure, reducing toxicity and stability. Heterologous expression of *mlrA* in different microorganisms may enhance its production and activity, promote additional knowledge on the enzyme, and support feasible applications. In this context, we intended to express the *mlrA* gene from *Sphingosinicella microcystinivorans* B9 in an industrial *Saccharomyces cerevisiae* strain as an innovative biological alternative to degrade microcystins. The *mlrA* gene was codon-optimized for expression in yeast, and either expressed from a plasmid or through chromosomal integration at the *URA3* locus. Recombinant and wild yeasts were cultivated in medium contaminated with microcystins, and the toxin content was analyzed during growth. Whereas no difference in microcystins content was observed in cultivation with the chromosomally integrated strain, the yeast strain hosting the *mlrA* expression plasmid reduced 83% of toxins within 120 h of cultivation. Our results show microcystinase A expressed by industrial yeast strains as a viable option for practical applications in water treatment.

## 1. Introduction

Cyanobacteria (blue-green algae) are prokaryotes well adapted for growth in eutrophic environments, which are able to produce compounds harmful to animals and humans, especially during massive growth or blooms [1]. Among its bioproducts, microcystins (MCs) are considered of greatest concern due to their prevalence and hepatotoxic effects [2]. Besides liver damage, exposure to MCs is associated with several other disorders in humans, depending on the duration and dose of exposure [3]. Moreover, microcystin-leucine-arginine (MC-LR) is the most potent variant among MC congeners (MC-LR, MC-RR, MC-YR, MC-LA, MC-LF), and it is classified as a possible carcinogen to humans by the International Agency of Research on Cancer [4,5]. Considering the risks posed by MC-contaminated water, the World Health Organization (WHO) set a provisional maximum concentration at 1.0 μg.L^−1^ MC-LR in drinking water, and a tolerable daily intake of 0.04 μg of MC-LR per kg of body mass per day for humans [6,7].

These toxins can pass through food webs, accumulating microcystins in fish tissue and agricultural crops, mainly in leafy vegetables irrigated with contaminated water [2,8]. The general structure of microcystins consists of a cyclic heptapeptide, which confers some stability to certain physical and chemical treatment processes [9]. In water treatment, methods usually applied have limited effectiveness for microcystin control, such as chlorination, UV, ozonation, carbon adsorption, and membrane filtration. Despite possible effects on MC removal or degradation, some of these physical and chemical processes may release other cytotoxins [10]. Therefore, new methods of controlling MCs that are effective, safe, and applicable in practice are needed.

**Figure 1:**
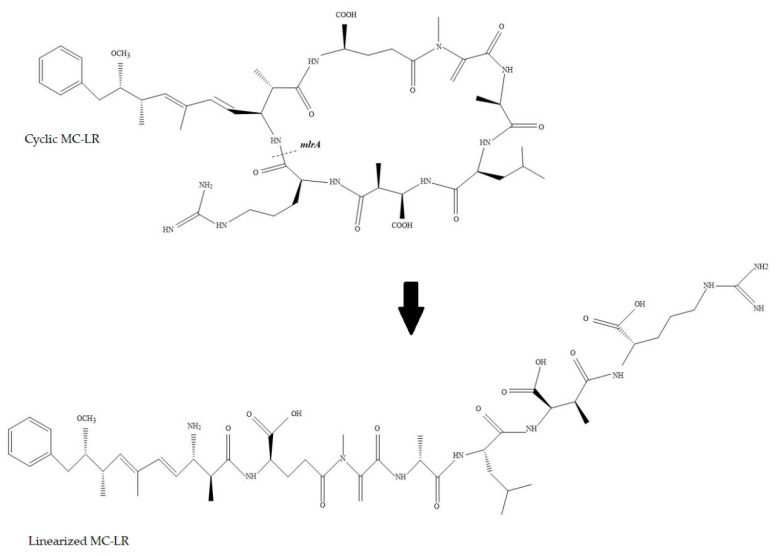
Linearization of Microcystin-LR by microcystinase A.

The MC degradation pathway led by microcystinases encoded by the *mlrABCD* complex is the most important and well-known biological tool for the environmental control of these toxins [11]. The first enzyme involved in MC degradation is encoded by the *mlrA* gene, cleaving the Adda–Arg bond in the MC-LR cyclic structure (Figure 1). Available data on the linear MC structure show toxicity is reduced approximately 2100-fold when compared to cyclic MC-LR [12,13]. In addition, the linearized toxin is less stable and active sites are more susceptible to enzymatic degradation [14]. Recent studies have focused on the heterologous expression of the *mlrA* gene in other bacterial species with the purpose of improving the efficiency of natural MC biodegradation systems. Dexter et al. (2021) described experiments that used this strategy and most instances obtained efficient degradation of MCs [15]. As the enzyme encoded by *mlrA* is highly effective and acts as a sustainable biological alternative to MCs degradation, its engineering and heterologous expression may lead to promising increased production and enhanced activity. Nevertheless, further studies are recommended to refine reported results through different technical approaches and to test alternatives to conventional expression in bacteria strains.

Yeasts are commonly manipulated by molecular engineering techniques aiming to increase the productivity of industrial strains or set new applications, mainly in food, chemical, and pharmaceutical areas. Among reported yeast species, *Saccharomyces cerevisiae* is the most widely studied due to its robustness and safety: it is classified as generally recognized as a safe (GRAS) microorganism [16]. Additionally, this microorganism grows in simple medium, providing high cell-density, and can produce post-translational modifications to expressed proteins, such as glycosylation [17]. These characteristics coupled with its well-characterized genetics motivate innovative academical and industrial applications of recombinant yeast [18]. 

Therefore, this work developed recombinant *S. cerevisiae* PE-2 strains for the heterologous expression of the *mlrA* gene from the *Sphingosinicella microcystinivorans* B9 strain. To date, this is the first attempt to express this gene in a widely used industrial yeast strain. The gene was codon-optimized for yeast expression and two different strategies were used: plasmid expression and chromosome integration of the *mlrA* gene into the *URA3* locus. Plasmid-hosting strains were selected considering their resistance to geneticin (G418). Gene integration into the yeast chromosome was confirmed by growth on selective media and by PCR amplification of the *mlrA* gene. The results demonstrated the feasibility of heterologous expression of microcystinases in yeast cells and provide a basis for further experiments to enhance strategies for controlling microcystins.

## 2. Materials and Methods

### 2.1. Strains, Plasmids and Reagents

*Saccharomyces cerevisiae* strains used in this work were: PE-2 (Brazilian diploid ethanol-producing yeast; Fermentec, Piracicaba, Brazil), YRH1624 (*MAT*alpha spore isolated from PE-2), and YRH1636 (*MAT*a spore isolated from PE-2). To generate haploid yeast strains YRH1624 and YRH1636, *S. cerevisiae* strain PE-2 was sporulated and 4-spore tetrads were dissected to YPD plates as described in Lopes et al. (2017) [19]. The mating type for each strain was determined as described in Section 2.7.

Expression plasmid pRH809 contains the *KanMX* marker for resistance to G418, the *Ashbya gossypii TEF* (*AgTEF*) promoter, and *S. cerevisiae RPL41B* terminator. The integration plasmid pRH806 contains the *AgTEF* promoter, multi-cloning site, and *S. cerevisiae ADH1* terminator, flanked by 400 to 500 bp of homology to the PE-2 *URA3* gene to target integration to the *URA3* locus. *Escherichia coli* competent NEB10β cells (NEB, Ipswich, MA, USA) were used for transforming and cloning the plasmids. Geneticin G418 (200 μg.mL^−1^), ampicillin (100 μg.mL^−1^), and chloramphenicol (100 μg.mL^−1^) were used for selection of microorganisms and prevention of contamination during cultivation. The restriction enzymes SpeI, SalI, XhoI, SacI, BssHII, and T4 DNA ligase were purchased from New England Biolabs (NEB; Ipswich, MA, USA).

*Sphingosinicella microcystinivorans* B9 strain was provided by Dr. Ken-Ichi Harada [20]. Tests previously performed by part of our research group confirmed this strain’s potential and high efficiency in MCs degradation. Additionally, *Microcystis* sp. TAC95 was cultivated and cyanobacterial extracts were prepared as described by Kuriama et al. (2012) [21]. Extracts were analyzed on HPLC-DAD and quantified by calibration curves prepared with standard MC-LR purchased from Sigma-Aldrich (St. Louis, MO, USA). Acetonitrile and ammonium acetate HPLC grade used on MCs analyses were purchased from Sigma-Aldrich (St. Louis, MO, USA) and JT Baker (Phillipsburg, NJ, USA), respectively.

In general, yeast strains were cultivated in YPD (1% yeast extract, 2% dextrose, 2% peptone), while *E. coli* were grown in LB medium (0.5% yeast extract, 1% tryptone, 1% NaCl). Selection of yeast strains with *mlrA* gene integrated to their chromosomes was performed with Yeast Nitrogen Base (YNB—Difco; Sparks, MD, USA) supplemented with 2% dextrose and amino acids (50 μg.mL^−1^ tyrosine, 50 μg.mL^−1^ serine, 50 μg.mL^−1^ valine, 50 μg.mL^−1^ isoleucine, 50 μg.mL^−1^ phenylalanine, 50 μg.mL^−1^ aspartic acid, 50 μg.mL^−1^ proline, 100 μg.mL^−1^ arginine, and 100 μg.mL^−1^ threonine). Uracil (175 μg.mL^−1^) and 5-fluoroorotic acid (1 mg.mL^−1^; 5-FOA; Zymo Research, Irvine, CA, USA) were added to YNB plates to select cells with mutation within the *URA3* locus.

### 2.2. Construction of the Expression Plasmid (pRH809+mlrA)

The *mlrA* expression vector (pRH809) has *AmpR* and *KanMX* markers, which confer ampicillin and geneticin (G418) resistance to the host, besides *AgTEF* promoter and *RPL41B* terminator regions. Codon-optimization of the *mlrA* gene for expression in *S. cerevisiae* cells was based on the *S. microcystinivorans* B9 strain genome deposited in GenBank (accession number AP018711) [20]. Adaptations were made manually based on a codon usage frequency table, and the final gene sequence was evaluated using an online tool for codon-optimization, resulting in an adequate expression level (www.genscript.com (accessed on 1 August 2019)).

A 1061 bp DNA fragment containing the codon-optimized *mlrA* gene and homology regions to the pRH809 vector was purchased from Fastbio (Ribeirão Preto, Brazil) and used to construct the expression plasmid pRH809+*mlrA*. pRH809 vector was digested with SpeI and SalI enzymes and the fragments were assembled using NEBuilder Hi-Fi DNA Assembly Kit (NEB) according to the developers’ instructions. The constructed expression plasmid was transformed into competent *E. coli* NEB10β cells following the developers’ protocols (NEB). Bacterial cloning was performed during overnight incubation at 37 °C and continuous agitation (250 rpm) in LB medium added with 100 μg.mL^−1^ of ampicillin. pRH809+*mlrA* plasmid was extracted on QIAPrep Spin MiniPrep Kit (Qiagen; Venlo, Netherlands), and the DNA concentration was measured on Nanodrop 2000/2000c Spectrophotometer (NanoDrop Technologies, Wilmington, DE, USA). The constructed expression plasmid had a fragment of its DNA amplified using M13 primers (M13F-5′GTAAAACGACGGCCAG3′; M13R-5′CAGGAAACAGCTATGAC3′), and analyzed by sequencing, confirming no mistakes in the assembled fragment. Part of the content of extracted pRH809+*mlrA* plasmid was used to transform PE-2 yeast cells, while the other was used to construct the integration vector.

### 2.3. pRH809+mlrA Transformation into PE-2 S. cerevisiae Strain

PE-2 yeasts were transformed using a standard lithium acetate method with some modifications [22]. Yeasts were grown in YPD medium overnight at 30 °C and constant agitation at 180 rpm. An aliquot was calculated and transferred to an additional flask to complete a 25 mL final volume of YPD medium, corresponding to the optical density (OD) of 0.1 at 600 nm. OD measurement was performed on the Anthos Zenyth 200 rt Spectrophotometer (Biochrom, Holliston, MA, USA). The yeast was incubated for 4 h at 30 °C and 220 rpm. Then, 10 mL of the medium was transferred to a 50 mL conical tube and centrifuged at 3500× *g* for 5 min. The supernatant was discarded and the pellet was suspended in 1 mL of a solution A (10X Tris-EDTA Buffer—TE, 1 mol.L^−1^ LioAc and water—1:1:8). The mixture was moved to a microcentrifuge tube and cells were pelleted at 10,000× *g* for 30 s. The supernatant was discarded and the process of suspension in solution A and centrifugation was repeated. Next, the pellet was suspended in 50 µL of solution A, and 1 μg of the plasmid (pRH809+*mlrA*) and 50 μg of DNA carrier (salmon sperm) were added. A total of 300 µL of solution B (10X TE Buffer, 1 mol.L^−1^ LioAc and 50% of polyethylene glycol—PEG—1:1:8) was added to the tube. The mixture was incubated at 30 °C for 30 min, 42 °C for 15 min, and kept at room temperature for 10 min. Then, the tube was centrifuged at 10,000× *g* for 1 min, the supernatant was discarded, and the pellet was suspended in 500 μL of sterile water. This last process was repeated once. Next, 100 µL of the suspended pellet was spread on YPD + G418 (200 μg.mL^−1^) agar plates and incubated for 48 h at 30 °C. Only transformed cells were able to grow in the presence of G418.

### 2.4. Construction of the Integration Vector

**Figure 2:**
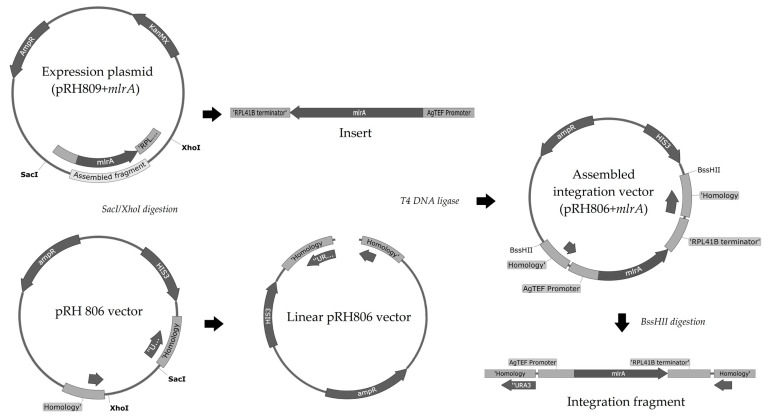
Construction map of the integration vector (pRH806+*mlrA*): Expression plasmid (pRH809+*mlrA*) was assembled as mentioned in the text (in Section 2.2). Both pRH809+*mlrA* and pRH806 vectors were digested with SacI and XhoI. Insert fragment (1901 bp) and the linear pRH806 vector (4610 bp) were ligated with T4 DNA Ligase. The assembled vector was digested with BssHII, and the integration fragment (2925 bp) was transformed into yeast cells. By crossing over with the homology regions, *mlrA* gene was integrated at the *URA3* locus.

The entire process to construct the integration vector is summarized in Figure 2. 

**Figure 3:**
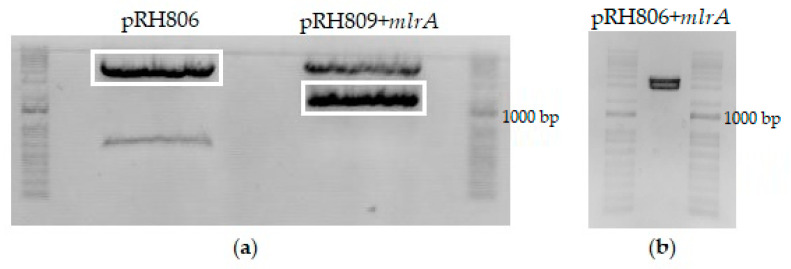
Electrophoresis analyses of procedures applied to construct the integration vector (pRH806+*mlrA*). (**a**)—SacI and XhoI digestion products from plasmids pRH809+*mlrA* (expression plasmid containing the *AgTEF* promoter-*mlrA*-‘*RPL41B* terminator) and pRH806 (vector to chromosomal integration). Bands marked containing 4610 bp and 1906 bp were excised, purified, and ligated to construct the new pRH806+*mlrA* plasmid. (**b**)—pRH806+*mlrA* vector digested with BssHII enzyme. Two DNA fragments were produced. The shortest fragment (2925 bp) contains *URA3* homology regions and the integrating expression cassette.

Originally, the pRH806 vector was constructed with an *AmpR* marker and two regions of homology to *S. cerevisiae URA3* locus. This plasmid was transformed and cloned into competent *E. coli* cells, extracted, and quantified as described previously for the pRH809+*mlrA* plasmid. Plasmids pRH806 and pRH809+*mlrA* were both digested with SacI and XhoI restriction enzymes. Fragments were analyzed by 0.8% agarose gel electrophoresis (Figure 3a). The longest pRH806 fragment (vector containing the *URA3* homology regions) and the shortest pRH809+*mlrA* fragment (insert with *mlrA* gene) were excised from the gel and purified with Pure Link Quick Gel Extraction and PCR Purification Combo Kit (Invitrogen, Waltham, MA, USA). The concentration of DNA in purified fragments was quantified on Nanodrop 2000/2000 c at 260 nm. The new pRH806+*mlrA* plasmid was assembled with T4 DNA ligase. The integration plasmid was transformed and cloned into *E. coli* competent cells, then extracted and quantified exactly as performed in the previous steps. The assembled vector (pRH806 plasmid with *URA3* homology region—*AgTEF* promoter—codon-optimized *mlrA*—*RPL41B* terminator—*URA3* homology region) was used to integrate the expression cassette into the *URA3* locus of *S. cerevisiae*. The vector was digested with the BssHII restriction enzyme in the regions of homology. To confirm fragment size and the efficiency of digestion, a 5 μL aliquot of the digestion product was analyzed in 0.8% agarose gel electrophoresis (Figure 3b). Two bands with a length of approximately 3000 bp confirmed the initial size of the plasmid and digestion by the enzyme. The remaining 75 μL from the restriction process were purified on Pure Link PCR Purification Kit (Invitrogen) and used to transform YRH1624 yeast cells.

### 2.5. Transformation into YRH1624 S. cerevisiae Strain and mlrA Integration into URA3 Locus

Transformation steps were carried out as described for pRH809+*mlrA*. After colonies grew on YPD agar plates, replica plates were performed on YNB+5-FOA+Uracil and YNB-Uracil plates. After 48 h at 30 °C, only *URA3* mutant colonies were able to grow in the presence of 5-FOA. Furthermore, *URA3* disrupted cells did not grow in the absence of Uracil supplementation. Then, colonies that grew on YNB+5-FOA+Uracil plates but did not grow on YNB-Uracil plates were selected and replicated on new plates to assess the mutation in *URA3* and the stability of such cells. The replication process was repeated 5 times. At the final of five replications, colonies considered recombinant at the *URA3* locus were confirmed by PCR amplification of the integrated *mlrA* gene.

The recombinant haploid YRH1624+*mlrA* strain (*MAT*alpha) was mated with YRH1636 wild-type strain (*MAT*a) as described by Treco and Winston (2008) [23]. Freshly grown yeasts from each strain were mixed on a YPD plate and incubated at 30 °C for 4 h. Then, the mixture was streaked on a YNB-Uracil plate and incubated at 30 °C for 48 h. YRH1636 wild-type and diploid strains were able to grow in the absence of uracil. Several colonies randomly selected were analyzed by PCR to confirm they were diploid and the integration of *mlrA* into their genome.

### 2.6. PCR Amplification and Identification of mlrA Integration

Integration of the *mlrA* gene into the *URA3* locus was confirmed by the amplification of a DNA fragment containing part of the integrated *mlrA* gene and a sequence outside of the homology region in the complementary strand of chromosome V. Primers were purchased from Integrated DNA Technologies (IDT, Coralville, IO, USA) and designed to amplify the 738 bp fragment. The assay was performed following instructions of developers with adjustments: mixture consisted of 1X Buffer, 5 mmol.L^−1^ MgCl_2_, 200 μmol.L^−1^ of each Deoxynucleotide triphosphates, 1 μmol.L^−1^ of each primer, 1.25 U of Dream Taq Hot Start DNA Polymerase (Thermo Scientific, Whaltam, MA, USA) and 1 μL of DNA as template. DNA extraction was performed on the PureLink Genomic DNA Mini Kit (Invitrogen). ddH_2_O was added up to 25 μL of final reaction volume. Sterile water was used as template for negative control. The amplification was carried out in a Veriti Thermal Cycler 9901 (Applied Byosistem, Thermo Scientific). Primer sequences and the amplification conditions are shown in Table 1. PCR products were analyzed by electrophoresis on 1.2% agarose gel stained with SYBR Safe DNA Gel Stain (Invitrogen).

### 2.7. Mating-Type PCR Testing

Yeast strains used for this experiment were tested to assess their mating type. PCR amplification confirmed the selection of opposite mating types to mate recombinant and wild-type strains, as well as selecting diploid recombinant colonies. One amplification reaction consisted of: mixture of 1X Buffer, 200 μmol.L^−1^ of each Deoxynucleotide triphosphates, 1 μmol.L^−1^ of each primer, 1.25 U of Gold Taq Hot Start Polymerase (Cellco; São Carlos, SP, Brazil), 1 μL of DNA as template and ddH_2_O up to 25 μL of final volume. Sterile water was added as template for negative control. The amplification process and primer sequences were shown in Table 1. Electrophoresis analysis of the PCR products were performed as described in the previous section for the *mlrA* gene integration.

### 2.8. MC Degradation Test

Prior to MC degradation tests, *Microcystis sp*. extracts were analyzed on HPLC-DAD and compared to standard curves performed with MC-LR. The retention time for both standard MC-LR and extracts peaks was the same. Possibly, most of the microcystin content in the extracts was composed of the MC-LR variant, however, no confirmation was carried out. Therefore, the toxins in extracts were regarded as MCs.

Initially, yeast strains were grown in YPD medium overnight at 30 °C and constant agitation at 180 rpm. Then, aliquots of grown medium were calculated considering an initial OD (600 nm) of 0.1 for 10 mL test volume. Calculated amounts were transferred to sterile tubes and centrifuged at 3500× *g* for 5 min. Supernatant was discarded and the pellet was resuspended in the degradation test medium (10 mL). One test sample consisted of 10 mL final volume containing: 1 mL of YPD, 10 μL of chloramphenicol (final concentration of 100 μg.mL^−1^), approximately 300 ng.mL^−1^ of equivalent MC-LR and sterile water. G418 (200 μg.mL^−1^) was added to cultivations with the expression plasmid (pRH809+*mlrA*). Tests were performed in triplicate. The incubation conditions were the same used for yeast growth: 30 °C and 180 rpm. Aliquots (600 μL) were collected from all the samples within 0, 24, 48, and 72 h of cultivation, and centrifuged at 10,000× *g* for 5 min. The supernatant was filtered on PVDF 0.22 μm, 13 mm hydrophilic syringe filters (Millex, Darmstadt, Germany) and stored at −20 °C until analyses on HPLC-DAD. The degradation test was performed with the three different recombinant strains, one wild-type strain, and a negative control (without any yeast). An additional test was performed at 120 h of cultivation with the strain host of the expression plasmid (pRH809+*mlrA*) and the wild type. Only these strains were selected for the extended test, since strains with chromosomally integrated *mlrA* were not able to degrade MCs on the preliminary tests.

### 2.9. HPLC-DAD Analyses of MCs

MCs analyses were performed on a Shimadzu HPLC System with a DGU-20As Degasser, LC-20AD Pump, CTO-20AC Oven, and a SPD-M20A Diode Array Detector (Shimadzu do Brasil, Barueri, SP, Brazil). Chromatographic separation was achieved at 40 °C using a Luna C18 column (Phenomenex; 4.6 × 250 mm × 5 μm). The mobile phase consisted of an isocratic mixture of 20 mmol.L^−1^ ammonium acetate and Acetonitrile (70:30; v:v). The flow rate was 1.0 mL.min^−1^ and 20 μL of samples were injected into the HPLC system. MCs were detected at their maximum absorbance of 238 nm. The full chromatographic run was 10 min and the peak corresponding to MCs was observed at 6.36 min. All samples were analyzed in duplicate.

### 2.10. Statistical Analyses

Analyses of variance (ANOVA) and Tukey test were performed using R Studio Version 2022.02.3 + 492 (www.rstudio.com (accessed on 5 September 2022)) to assess differences on microcystin mean concentrations with tested yeasts. Assays were performed in triplicate and a 95% confidence interval (*p* < 0.05) was used on statistical analysis.

## 3. Results

The *mlrA* gene used in this study originated in the bacterial strain *S. microcystinivorans* B9. The gene was codon-optimized for expression in *S. cerevisiae. S. microcystinivorans* B9 naturally has the *mlr* gene cluster in its genome, and it was made available for tests carried out by part of our research group. Although available reports have demonstrated great potential for cyanotoxins degradation [21], to date, few studies are available on the evaluation of this bacterium to control MCs, and the heterologous expression of *mlrA* from the B9 strain has not been assessed previously. Therefore, the *mlrA* gene from *S. microcystinivorans* B9 was selected to be expressed for the first time in an industrial *S. cerevisiae* strain (PE-2) using both plasmid-based expression and chromosomal integration.

**Figure 4:**
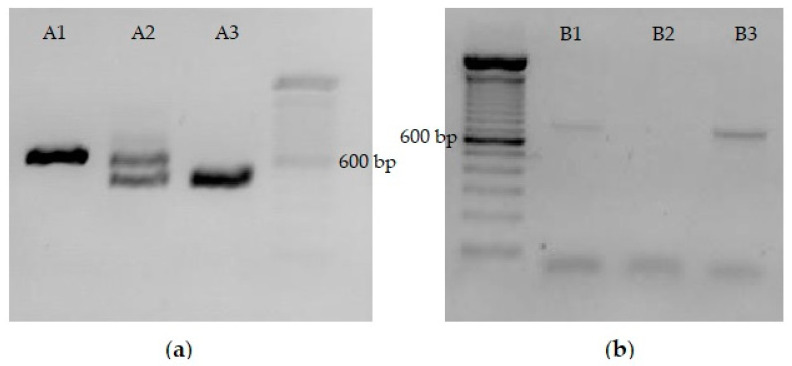
Electrophoresis analyses assessing mating type and *mlrA* integrated gene on yeasts (**a**) Mating type of tested yeasts: A1—haploid wild-type YRH1636 (*MAT*a); A2—diploid strain obtained from YRH1624 recombinant strain mating with YRH1636 wild type; A3—haploid integrated YRH1624 strain (*MAT*alpha). (**b**) *mlrA* gene PCR amplification: B1—diploid+*mlrA* integrated strain; B2—YRH1636 (wild type); B3—YRH1624+*mlrA* integrated strain.

The *S. cerevisiae* strain PE-2 has been shown to have multiple mutations in the *HO* endonuclease gene, which is required for mating type switching, likely rendering it non-functional [24]. To generate haploid strains of PE-2, the diploid PE-2 strain was first sporulated, and individual spores were isolated and tested to determine their mating type and stability of the mating type. Strains YRH1624 and YRH1636, haploids derived from PE-2, were shown to maintain stable *MAT*alpha and *MAT*a mating types, respectively, indicating that the *HO* endonuclease in strain PE-2 is not functional (Figure 4a). YRH1624 strains with integrated *mlrA* were then successfully obtained and collected after repeated cultivation on selective medium (as described in Section 2.5). Integration of the *mlrA* gene into the *URA3* locus was confirmed by PCR amplification. Next, recombinant *MAT*alpha colonies were mated with YRH1636 wild-type (*MAT*a) to obtain a recombinant diploid. After mating, colonies were randomly selected to assess mating type and presence of *mlrA* at the *URA3* locus (Figure 4). Diploid strains with integrated *mlrA* were then used in MC degradation tests. 

The recombinant yeast strains and a YRH1636 wild-type strain were first used to test MC degradation during 72 h of cultivation. Data are shown in Table 2. As expected, no difference was observed in the concentration of MCs over time for the negative control and wild-type strain, however, none of the recombinant strains with chromosomally integrated *mlrA* reduced the toxins’ content. On the other hand, the yeast strain hosting the *mlrA* expression plasmid (pRH809+*mlrA*) reduced MC concentration significantly, even after only 24 h of cultivation.

**Figure 5:**
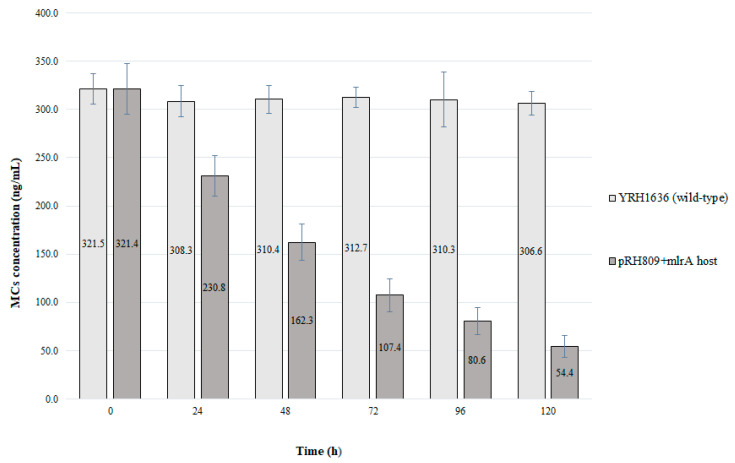
Time course of MC degradation test. Negative control consisted of the wild-type yeast (YRH1636). For the negative control, there was no difference among the mean concentrations of MCs during all the time of cultivation. For the strain with pRH809+*mlrA*, MC content differed among all the analyzed times (*p* < 0.05).

Although the strains with integrated *mlrA* did not reduce MC concentration, the approach using the expression plasmid was efficient. Within the first 24 h of growth MC concentration in the medium was reduced by 32.2%. At the end of 72 h, the total MC content was reduced by 69.6%. As only the PE-2 strain hosting the pRH809+*mlrA* plasmid showed the ability to degrade MCs, an extended test with this strain was performed for 120 h of cultivation and the recombinant yeast was compared to the wild type (Figure 5).

Prolonged cultivation of the strain with pRH809+*mlrA* reinforced the efficient reduction in the concentration of MCs. During the first 24 h of cultivation, toxin content was reduced by 28.2%. After 120 h of cultivation, about 267 ng.L^−1^ of MCs were reduced from the medium, which represents degradation of more than 83% of total MCs. The wild-type yeast strain did not reduce the concentration of MCs from the medium. 

**Figure 6:**
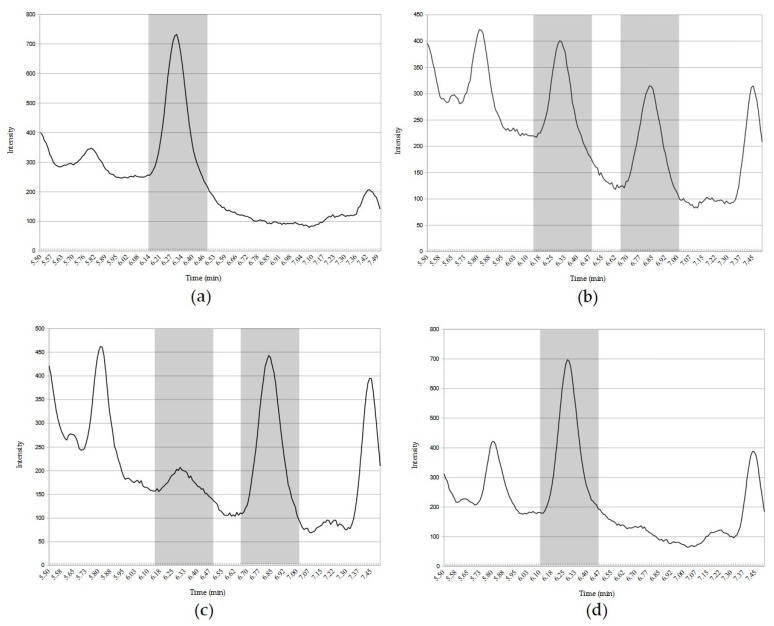
Combined chromatograms of yeast strains cultivated with 300 ng.mL^−1^ MCs in medium. Highlighted peak at 6.36 min represents MCs extracts confirmed by comparison with MC-LR standard. The second highlighted peak area on 6.87 min is suggested to be the linearized MC. (**a**)—initial point/YPD medium added with MCs before yeast inoculation (0 h); (**b**)—recombinant yeast (PE-2+pRH809+*mlrA*) culture extracts after 48 h of cultivation; (**c**)—recombinant yeast (PE-2+pRH809+*mlrA*) culture extracts after 120 h of cultivation; (**d**)—wild yeast (YRH1636) culture extract after 120 h of cultivation.

In addition to the reduction in MC concentrations observed by our results, analyses of the chromatograms suggested that part of the original cyclic toxin was being transformed into linearized MC. Chromatograms of the recombinant and wild-type yeasts were combined from 0 to 120 h of cultivation. In Figure 6, HPLC-DAD results are shown for elution times from 5.5 to 7.5 min.

At 6.36 min, there was a peak corresponding to cyclic MC, which was confirmed with a MC-LR standard. During the cultivation of the recombinant yeast (PE-2+pRH809+*mlrA*), a significant reduction in the peak area was observed at 6.36 min and a new peak appeared at 6.87 min. Considering the expected activity of microcystinase A, it is suggested that this new peak consists of linearized MCs. The area of the new peak at 6.87 min (with 120 h of cultivation) was quantified as 246.7 ng.mL^−1^ on the MC-LR calibration curve. This concentration added to the remaining 55.4 ng.mL^−1^ of cyclic MCs sums to 302.1 ng.mL^−1^, which is very close to the MCs concentration at the beginning of the test. Chromatograms from wild-type yeast and yeast with integrated *mlrA* showed no significant difference in the 6.36 min peak area. Moreover, no peak was observed at 6.87 min for these strains.

## 4. Discussion

Two strategies were used by this study to heterologously express the *mlrA* gene in *S*. *cerevisiae*: plasmid-based expression and chromosomal integration. The first tested approach was successful and resulted in a significant decrease in MC content. On the other hand, the integrated recombinant strains did not show degradation activity when cultured with MCs. Chromosomal integration of target genes is a strategy used to overcome possible problems of plasmid-based overexpression. Advantages of chromosomal integration include increased stability of expression and eliminating the need for antibiotics used for plasmid maintenance [25]. However, no MC degradation was observed for strains with integrated *mlrA*. 

PCR amplification of the *mlrA* gene in selected strains indicated that the *mlrA* gene was correctly integrated at the *URA3* locus (Figure 4). One possibility for the lack of expression is the orientation of integrated *mlrA* gene. Previous studies reported transcriptional interferences in *S. cerevisiae* resulting from promoter occlusion or transcriptional collision [26,27]. Steiner and Philippsen (1994) described that the *AgTEF* promoter contains two upstream activation sequences (UASrpg) that bind Rap1p, a protein that has been shown to act as a roadblock to upstream transcription by inducing its termination [28]. Due to the presence of Rap1p binding sites in the *AgTEF* promoter, and its orientation to the remaining *URA3* gene, promoter occlusion is not likely. Conversely, the orientation of the integrated *mlrA* gene with respect to the *URA3* promoter suggests that transcript collision could be the cause of poor expression. Another hypothesis for the inability of the strain with integrated *mlrA* to degrade MCs is that the gene copy number compared to the plasmid-based strain was too low. Nonetheless, to date, it has not been possible to define exactly what is causing the inhibition of *mlrA* expression in integrated strains or their inability to reduce MCs content. Further studies should be carried out to assess the possibilities that might be affecting the gene expression, in addition to considering solutions, such as using different promoters, changing integration orientation, increasing the copy number of integrated gene, and targeting a different locus for *mlrA* chromosomal integration.

Whilst yeast with integrated *mlrA* did not reduce MCs, efficient degradation of the toxin was observed for cells hosting the expression plasmid (pRH809+*mlrA*). These results reinforce the ability of microcystinase A to be expressed in *S. cerevisiae* cells and indicate the enzyme’s potential to degrade such contaminants. The objective of this work was to provide a novel biological method to reduce MC toxicity and its impact on public health. The *mlrA* gene encodes the first enzyme of the cluster *mlrABCD* related to the natural degradation of microcystins. This enzyme hydrolyzes the Adda–Arg bond in cyclic structure of MC-LR, reducing its stability and toxicity [14]. A codon-optimized version of the *mlrA* gene sequence from *S. microcystinivoran*s B9 strain was used in our study. *S. microcystinivorans* B9 has proved to be efficient in the natural degradation of microcystins, as well as showing the degradation of MCs with immobilized bacteria, and further activity for different amino acid-containing compounds during bacterial growth [29,30,31]. Other studies confirmed the potential of MC degradation by B9 strain as one of the most efficient among tested microorganisms, reducing 95% of the initial concentration of toxins (1.0 μg.mL^−1^) after 96 h of cultivation [20]. Despite the reported positive results for such bacteria, it is important to note that the genome of *S. microcystinivorans* B9 has other genes involved in the degradation of MCs. Thus, to study *mlrA* activity in the degradation of MCs, and enhance its potential for further applications, engineering and expressing it in different biological systems is required. 

Different strategies are available for expressing the *mlrA* gene, presenting distinct potentials of enzyme production and activity. For example, Dexter et al. (2018) expressed the gene in cyanobacteria, resulting in an increased production of the enzyme compared to the native bacteria that hosts *mlrA* and prolonged enzymatic activity under semi-natural conditions of water contamination [32]. Wang et al. (2017) expressed the *mlrA* gene from *Novosphingobium sp*. THN1 in *E. coli* and evaluated its enzyme activity on microcystin–arginine–arginine (MC-RR) degradation. The tests were performed with intact recombinant cells and cell-free extracts. After 56 h of incubation, both treatments reduced more than 70% of MC-RR, however, different trends were observed. Cell-free extracts reduced microcystin concentration at an almost constant rate, while the intact bacteria degraded 73.8% in just 8 h. After this period, the degradation rate was reduced, and a plateau was reached. It was suggested that with the lower concentration of MCs in the medium, the enzyme activity was also reduced [33]. A similar degradation plateau was also seen in a study using probiotic bacteria to remove MC-LR. Using live bacteria, Nybom et al. (2007) showed a maximum MC reduction of approximately 70% with most conditions tested resulting in 40% of MC-LR removed [34]. In each case, the degradation slowed significantly after the first 5 h. This behavior was not observed in our work, in which the reduction in MCs occurred at an almost constant rate of 30% per day and reached 83%.

Heterologous expression of the *mlrA* gene has been performed in *E. coli* strains by most of the available research. Zhu et al. (2016) expressed the *mlrA* gene from *Rhizobium sp*. in *E. coli* cells and 8.3 μg.L^−1^ of MC-LR was degraded within 10 h of incubation following lag phase [35]. On the other hand, Moolwang et al. (2021) worked with purified *mlrA* recombinant enzyme expressed in *E. coli* BL21 strains. A complete degradation of 30 μg.mL^−1^ of MC-LR was reported within 30 h of incubation [36]. In addition, Liu et al. (2020) overexpressed the *mlrA* gene in K12 B1 strain reducing intracellular and extracellular MC content. Furthermore, *Microcystis aeruginosa* growth in BG11 medium was inhibited, which represents a useful biological tool in the control of toxigenic cyanobacteria [37]. In all the studies mentioned above, expression of the *mlrA* gene was induced with IPTG (isopropyl-β-D-thiogalactoside), and experiments reported by Moolwang et al. (2021) and Liu et al. (2020) were performed with the purified enzyme [35,36,37]. In contrast, here, a constitutive promoter was used, which does not require adding a reagent for induction and MC degradation was assessed during yeast growth.

Genetic engineering aiming to couple the efficient biodegradation of toxins by recombinant enzymes with desired characteristics of yeasts represents a promising alternative to the future development of practical applications for the *mlrA* enzyme. Working with *S. cerevisiae* strains has several advantages that can result in significant advances for research and industrial purposes. The yeast is considered a valuable model organism for genetic engineering due to its highly efficient metabolism, pH tolerance, safety, ease of cultivation, and resistance to some toxic compounds, including MC-LR [38]. Furthermore, as it is the most well-studied eukaryotic microorganism, numerous genetic tools are extensively described for *S. cerevisiae* engineering [19,39].

To date, only one report was found regarding the heterologous expression of an *mlrA* gene in *S. cerevisiae*. Broman et al. (2017) evaluated the expression of a codon-optimized *mlrA* gene from Sphingomonas sp. ACM-3962 in yeast cells. The *GAL1* promoter was used and expression was induced with 2% galactose. Enzymes were purified and applied to MC-LR degradation tests. Enzymatic activity was observed as a decrease in MC-LR intensity over time and results confirmed the possibility of engineering *mlrA* and expressing it in yeast cells [40]. Nevertheless, it is important to highlight fundamental differences in the conceptualization and objectives of both studies. The strategies here used a constitutive promoter, and optionally a gene chromosomal integration aiming to provide constant enzyme production and improve stability of the cells. Additionally, the enzymatic activity was measured by the reduction in MCs concentration during yeast cultivation. In addition, this is the first time that a widely used industrial *S. cerevisiae* strain was applied to express a *mlrA* gene.

In the absence of microcystinase A, glutathione and cysteine conjugates of MC are common metabolites seen as the first step in detoxification of cyclic MC [41]. Since shifted or decreased MCs peaks were not observed in cultures with the negative control and wild yeast (Figure 6 d), we do not believe significant levels of GSH-MCs were formed with our strains. The activity of microcystinase A expressed in yeast was observed as the reduction in MC content (Figure 6). A new peak appearing only in cultures of the yeast hosting the *mlrA* expression plasmid likely represents the enzymatic product (i.e., linear MCs). Other studies support this hypothesis, such as Fionah et al. (2022) in which the appearance of a peak corresponding to linearized MC occurred after the expression of *mlrA*: the identity of the new compound as linear MC was confirmed by LC-MS [42]. A new peak in microcystinase assays was also reported by Cai et al. (2022). After LC-MS analyses, it was confirmed to be linearized MC [43]. Although the discussion above strongly suggests that the new peak observed in our study is linearized MCs, it was not confirmed by secondary analysis.

Alternatively, the linearization of cyclic MC can be associated to different physical and chemical methods. The association of microcystinase A activity with H_2_O_2_ treatment, reduced simultaneously the toxicity of MC and the growth of cyanobacteria [44]. Wu et al. (2019) immobilized the enzyme in L-cystein graphene oxide and observed the degradation of 83% of nodularins within just one hour of incubation [45]. These are reported alternatives that can be used to spread *mlrA* applicability and increase the degradation of cyanotoxins. Here, MCs were solely treated with the *mlrA* enzyme, so our results offer proof of concept that *S. cerevisiae* is an appropriate host to produce active microcystinase A.

## 5. Conclusions

Expression of the codon-optimized *mlrA* gene in *S. cerevisiae* strains was assessed by two different strategies: using an expression plasmid and gene integration at the *URA3* locus. Chromosomal integration of the *mlrA* gene at the *URA3* locus was successfully achieved, and both haploid and diploid strains were generated with the integrated gene. Although presence of the gene was confirmed, MCs were not degraded by these strains. However, the PE-2 based strain containing the expression plasmid (pRH809+*mlrA*) was highly efficient at degrading MCs, reducing MC concentration by 83% after 120 h. The results demonstrate that industrial *S. cerevisiae* strains can be used as host for synthesizing microcystinase A. Further studies are warranted to improve the activity of the recombinant enzyme and define suitable methods for its practical application.

## Figures and Tables

**Table 1:** Primers sequences and PCR conditions to assess *mlrA* gene on integrated yeast strains and mating type *.

*MlrA* Fragment Amplification		
**Primers**	**Sequences**	
*mlrA* F	5′ TTA TGG TGA AGG ATA AGT TTT GAC C 3′	
*mlrA* R	5′ TTA GTT TGC CGC TAT GCC TC 3′	
**PCR Conditions**		
	Initial denaturation	95 °C/3 min
35 cycles	Denaturation	95 °C/30 s
	Annealing	50 °C/1 min
	Extension	72 °C/1 min
	Final extension	72 °C/10 min
**Mating type**		
**Primers**	**Sequences**	
*MAT*a	5′ ACT CCA CTT CAA GTA AGA GTT TG 3′	
*MAT*alpha	5′ GCA CGG AAT ATG GGA CTA CTT CG 3′	
*MAT*locus	5′ AGT CAC ATC AAG ATC GTT TAT GG 3′	
**PCR Conditions**		
	Initial denaturation	95 °C/10 min
35 cycles	Denaturation	95 °C/30 s
	Annealing	50 °C/30 s
	Extension	72 °C/1 min
	Final extension	72 °C/2 min

* PCR amplification of the *mlrA* integrated gene was performed with Dream Taq Hot Start DNA Polymerase (Thermo Scientific), while Mating type amplification was performed with Gold Taq Hot Start Polymerase (Cellco). PCR products were analyzed on 1.2% agarose gel electrophoresis.

**Table 2:** MCs degradation test during 72 h of yeast growth *.

Time (h)	Microcystins Concentration (Mean ± Standard Deviation—ng.mL^−1^)				
	Negative Control	Wild-Type Yeast	pRH809+*mlrA* Host	Integrated Strain (n)	Integrated Strain (2n)
0	295 ± 12.9 ^Aa^	295 ± 12.9 ^Aa^	295 ± 12.9 ^Aa^	295 ± 12.9 ^Aa^	295 ± 12.9 ^Aa^
24	298 ± 10.9 ^Aa^	291 ± 17.3 ^Aa^	200 ± 70.0 ^Bb^	286 ± 18.7 ^Aa^	285 ± 25.9 ^Aa^
48	300 ± 21.4 ^Aa^	299 ± 18.5 ^Aa^	153 ± 46.7 ^Bc^	285 ± 17.0 ^Aa^	290 ± 17.7 ^Aa^
72	292 ± 14.4 ^Aa^	294 ± 19.2 ^Aa^	90 ± 50.6 ^Bd^	284 ± 11.5 ^Aa^	286 ± 22.8 ^Aa^

* Different uppercase letters mean differences in row (by yeast strains). Different lowercase letters mean differences in column (on time). Negative control was performed without any yeast strain. Wild-type is YRH1636 yeast. PE-2 hosts the expression plasmid (pRH809+*mlrA*). Haploid (n) integrated strain is YRH1624+*mlrA*. Diploid (2n) integrated strain was obtained from YRH1624+*mlrA* mating with wild-type YRH1636.

## Data Availability

The data presented in this study are available on request from the corresponding author.
